# Memory Inflation Drives Tissue-Resident Memory CD8^+^ T Cell Maintenance in the Lung After Intranasal Vaccination With Murine Cytomegalovirus

**DOI:** 10.3389/fimmu.2018.01861

**Published:** 2018-08-14

**Authors:** Kaitlyn M. Morabito, Tracy J. Ruckwardt, Erez Bar-Haim, Deepika Nair, Syed M. Moin, Alec J. Redwood, David A. Price, Barney S. Graham

**Affiliations:** ^1^Viral Pathogenesis Laboratory, Vaccine Research Center, National Institute of Allergy and Infectious Diseases, National Institutes of Health, Bethesda, MD, United States; ^2^Department of Microbiology and Immunology, Georgetown University Medical Center, Washington, DC, United States; ^3^Department of Biochemistry and Molecular Genetics, Israel Institute for Biological Research, Ness-Ziona, Israel; ^4^Institute for Immunology and Infectious Diseases, Murdoch University, Perth, WA, Australia; ^5^Division of Infection and Immunity, Cardiff University School of Medicine, Cardiff, United Kingdom; ^6^Human Immunology Section, Vaccine Research Center, National Institute of Allergy and Infectious Diseases, National Institutes of Health, Bethesda, MD, United States

**Keywords:** CD8^+^ T cells, cytomegalovirus, memory inflation, respiratory syncytial virus, tissue-resident memory, vaccine

## Abstract

Tissue-resident memory T (T_RM_) cells provide first-line defense against invading pathogens encountered at barrier sites. In the lungs, T_RM_ cells protect against respiratory infections, but wane more quickly than T_RM_ cells in other tissues. This lack of a sustained T_RM_ population in the lung parenchyma explains, at least in part, why infections with some pathogens, such as influenza virus and respiratory syncytial virus (RSV), recur throughout life. Intranasal (IN) vaccination with a murine cytomegalovirus (MCMV) vector expressing the M protein of RSV (MCMV-M) has been shown to elicit robust populations of CD8^+^ T_RM_ cells that accumulate over time and mediate early viral clearance. To extend this finding, we compared the inflationary CD8^+^ T cell population elicited by MCMV-M vaccination with a conventional CD8^+^ T cell population elicited by an MCMV vector expressing the M2 protein of RSV (MCMV-M2). Vaccination with MCMV-M2 induced a population of M2-specific CD8^+^ T_RM_ cells that waned rapidly, akin to the M2-specific CD8^+^ T_RM_ cell population elicited by infection with RSV. In contrast to the natural immunodominance profile, however, coadministration of MCMV-M and MCMV-M2 did not suppress the M-specific CD8^+^ T cell response, suggesting that progressive expansion was driven by continuous antigen presentation, irrespective of the competitive or regulatory effects of M2-specific CD8^+^ T cells. Moreover, effective viral clearance mediated by M-specific CD8^+^ T_RM_ cells was not affected by the coinduction of M2-specific CD8^+^ T cells. These data show that memory inflation is required for the maintenance of CD8^+^ T_RM_ cells in the lungs after IN vaccination with MCMV.

## Introduction

Tissue-resident memory T (T_RM_) cells protect against invading pathogens in barrier tissues by direct killing of infected cells and by recruitment of other immune effector cell populations into the tissue. Much work has been done in recent years to characterize the migration pattern, function, and phenotype of T_RM_ cells in various anatomical locations (1–4). It has become clear that T_RM_ cells are heterogeneous, and that the requirements for localization and maintenance differ across tissues (4–9). In the lungs, T_RM_ cells have been shown to mediate immune protection against respiratory syncytial virus (RSV) (10–12) and heterosubtypic cross-protection against influenza virus (13–16). T_RM_ cells are also important for immune protection against cancer (17–23). In particular, T_RM_ cells have been shown to enhance the efficacy of intranasally administered cancer vaccines in mouse orthotopic head and neck tumor models (23). The abundance of T_RM_ cells in malignant lung tumors further correlated with survival in humans (23). However, lung-resident T_RM_ cells tend to wane over time, potentially reflecting a harsher and more dynamic environment compared with other barrier tissues (13, 14, 16, 24, 25). This progressive loss of T_RM_ cells likely explains why recurrent infections with RSV and influenza virus occur throughout life. Vaccination strategies aimed at maintaining high levels of T_RM_ cells in the lungs may therefore enhance immunity against respiratory pathogens and cancers.

Cytomegalovirus (CMV) has been shown to elicit robust populations of T_RM_ cells in some tissues (26, 27). The persistent nature of CMV leads to a unique phenomenon among memory CD8^+^ T cells, which has been well characterized in mouse models using murine cytomegalovirus (MCMV). Specifically, MCMV infection generates two distinct populations of memory CD8^+^ T cells, termed conventional and inflationary (28–32). Conventional CD8^+^ T cell populations expand during acute infection and then contract, whereas inflationary CD8^+^ T cell populations, which may not predominate in the early phase, continue to accumulate over time within the effector memory (EM) compartment. The ability to drive memory inflation may explain why CMV vectors have shown promise as vaccine candidates, protecting against various cancers and infectious agents and providing effective immunocontraception (33–41).

Several factors determine whether a particular epitope will elicit conventional or inflationary CD8^+^ T cell populations. For inflationary memory responses, the source protein must be transcribed during latency, a feature that depends primarily on location within the genome (42). In addition, the derived epitope may require processing by constitutive proteasomes, because antigen presentation occurs predominantly on the surface of non-hematopoietic cells, which lack immunoproteasomes (43, 44). Interclonal competition may also play a role, given the observation that high-avidity clonotypes are preferentially selected for inflation during MCMV infection (45–47). Similar findings have been reported in the setting of human CMV infection (41, 48–50). Other potential contributors include epitope-dependent requirements for co-stimulation and CD4^+^ T cell help (51–55). Memory inflation is therefore difficult to predict, even in well-defined mouse models, yet a detailed understanding of this phenomenon is critical for the design of effective vaccines that deliver protective antigens vectored by CMV.

Infection of CB6F1 mice with RSV elicits CD8^+^ T cell responses that reproducibly target an immunodominant epitope from the M2 protein (K^d^/M2_82–90_) and a subdominant epitope from the M protein (D^b^/M_187–195_) (56). The M-specific CD8^+^ T cell population typically incorporates high-avidity clonotypes expressing private T cell receptors with characteristic sequence motifs, leading to greater levels of cytokine production and more effective killing of virus-infected targets in side-by-side comparisons with the M2-specific CD8^+^ T cell population (57–59). In addition, M-specific CD8^+^ T cells regulate the magnitude of the otherwise numerically dominant M2-specific CD8^+^ T cell population, an effect that mitigates the immunopathology associated with acute RSV infection (57).

Intranasal (IN) vaccination with an MCMV vector expressing the M protein of RSV (MCMV-M) has been shown to generate a robust population of M-specific CD8^+^ T_RM_ cells with an effector/EM phenotype and augment early viral control relative to vaccination with MCMV alone or MCMV-M inoculated *via* the intraperitoneal (IP) route (60). In this study, we characterized the M2-specific CD8^+^ T cell response to IN vaccination with an MCMV vector expressing the M2 protein of RSV (MCMV-M2). Vaccination with MCMV-M2 induced a population of M2-specific CD8^+^ T_RM_ cells in the lungs that subsequently waned over time, whereas vaccination with MCMV-M induced a population of M-specific CD8^+^ T_RM_ cells in the lungs that subsequently inflated over time. Coadministration of both vaccines diminished the M2-specific CD8^+^ T cell response, but not the M-specific CD8^+^ T cell response, during the acute phase of infection, but had no impact on the magnitude of the conventional M2-specific CD8^+^ T cell population or the inflationary M-specific CD8^+^ T cell population during the chronic phase of infection. Moreover, the inclusion of MCMV-M2 neither enhanced nor impaired the protective effects of vaccination with MCMV-M alone in challenge experiments with RSV.

## Materials and Methods

### Mice

All experiments were conducted with age-matched (6–10 weeks) female CB6F1/J mice (Jackson Laboratories, Bar Harbor, ME, USA). Mice were maintained under specific-pathogen-free conditions on standard rodent chow and water supplied *ad libitum* in the Animal Care Facility at the National Institute of Allergy and Infectious Diseases. This study was carried out in accordance with the recommendations and guidelines of the NIH Guide to the Care and Use of Laboratory Animals. The protocol was approved by the Animal Care and Use Committee of the Vaccine Research Center, National Institute of Allergy and Infectious Diseases, National Institutes of Health. Mice were housed in a facility fully accredited by the Association for Assessment and Accreditation of Laboratory Animal Care International (AAALAC). Animal procedures were conducted in strict accordance with all relevant federal and National Institutes of Health guidelines and regulations.

### Cell Lines

CB6F1 mouse embryonic fibroblasts (MEFs) were isolated as described previously (60). MEFs were cultured in Advanced Dulbecco’s Modified Eagle’s Medium (DMEM; Invitrogen, Carlsbad, CA, USA) containing 10% fetal bovine serum (FBS), 2 mM glutamine, 10 U/ml penicillin G, 10 µg/ml streptomycin sulfate, and 0.1 M HEPES (DMEM-10). Human epithelial type 2 (HEp-2) cells were cultured in Eagle’s Minimal Essential Medium (MEM; Invitrogen) containing 10% FBS, 2 mM glutamine, 10 U/ml penicillin G, 10 µg/ml streptomycin sulfate, and 0.1 M HEPES (MEM-10).

### Viruses and Infection

Recombinant MCMVs were made using a bacterial artificial chromosome (BAC) system as described previously (35). Briefly, the M and M2 proteins from RSV were inserted into the IE2 gene of the K181Δm157 strain of MCMV using two-step allele replacement. BACs were extracted from *E. coli* using a NucleoBond Xtra Maxi Prep Kit (Clontech, Mountain View, CA, USA). MEFs were transfected with recombinant BACs by calcium phosphate precipitation (Clontech) as described previously (35). Single plaques were isolated by serial dilution after viral passage and selected based on excision of the BAC cassette determined by loss of GFP and confirmed by PCR. Viral stocks were made by sonication of infected MEFs, and plaque assays were performed in triplicate on CB6F1 MEFs. Mice were vaccinated IN with 3 × 10^5^ PFU of recombinant MCMV-M and/or MCMV-M2 in 100 µl of DMEM-10 under isoflurane anesthesia (3%). For RSV challenge, stocks were generated from the A2 strain by sonication of infected HEp-2 monolayers as described previously (61). Mice were challenged IN with 2 × 10^6^ PFU of RSV in 100 µl of MEM-10 under isoflurane anesthesia (3%). All mice were euthanized *via* the administration of pentobarbital (250 mg/kg).

### Intravascular Staining and Flow Cytometry

Mice were injected intravenously (IV) with 3 µg of anti-CD45 (BD Biosciences, San Jose, CA, USA). Five minutes after intravascular staining, mice were euthanized with pentobarbital, and the lungs were harvested at various time points. Lymphocytes were isolated by physical disruption of tissue using a GentleMACs Machine (Miltenyi Biotec, San Diego, CA, USA) and separated using density gradient centrifugation with Fico-LITE (Thermo Fisher Scientific, Waltham, MA, USA). Isolated mononuclear cells were washed with phosphate-buffered saline (PBS) and resuspended in fluorescence-activated cell sorting buffer (PBS supplemented with 1% FBS and 0.05% sodium azide). Cells were stained with directly conjugated antibodies specific for the lineage markers CD3 (145-2C11) and CD8 (53-6.7) (BD Biosciences) and the memory markers CD44 (IM7), CD62L (MEL-14), CD127 (A7R34), KLRG1 (2F1/KLRG1), CD69 (H1.2F3), and CD103 (M290) (BD Biosciences or BioLegend, San Diego, CA, USA). Dead cells were excluded from the analysis using LIVE/DEAD Fixable Aqua (Invitrogen). Antigen-specific CD8^+^ T cells were identified using D^b^/M_187–195_ (RSV M) or K^d^/M2_82–90_ (RSV M2) tetramers (MBL, Woburn, MA, USA). For validation of intravascular staining, cells were labeled with directly conjugated antibodies specific for CD3 (145-2C11), CD11c (N418), CD64 (X54-5/7.1), SiglecF (E50-2440), and CD11b (M1/70) (BD Biosciences or BioLegend). Data were acquired using an LSR II flow cytometer (BD Biosciences) and analyzed with FlowJo software version 9.9.6 (TreeStar, Ashland, OR, USA). Memory phenotypes were further analyzed using Pestle version 1.6.2 and SPICE version 6.0 (http://exon.niaid.nih.gov/spice/).

### ONX-0914 Inhibition Study

Mice were treated subcutaneously on days 0, 2, 4, and 6 with 2, 6, or 10 mg/kg of ONX-0914 (PR-957; Selleck Chemical, Houston, TX, USA) or vehicle control (10% captisol in 10 mM sodium citrate). On day 0, mice were infected IN as described above with 2 × 10^6^ PFU of RSV. On day 7, mice were euthanized with pentobarbital, and the lungs were harvested and processed as described above.

### Plaque Assay

Lungs were weighed and quick-frozen in 10% MEM-10, and plaque assays were performed as described previously (62). Briefly, thawed lung tissue was dissociated using a GentleMACs Machine (Miltenyi Biotec). Cell suspensions were pelleted to remove cellular debris, and clarified supernatants were serially diluted and inoculated in triplicate on 80% confluent HEp-2 cell monolayers. After rocking for 1 h at room temperature, monolayers were overlaid with 0.75% methyl cellulose in MEM-10 and incubated at 37°C. Cells were fixed with 10% buffered formalin and stained with hematoxylin and eosin on day 4. Plaques were counted and expressed as Log_10_ PFU/g of lung tissue. The limit of detection was 1.8 Log_10_ PFU/g.

### Statistical Analysis

Statistical analyses were performed using a one-way or two-way ANOVA as appropriate for multiple comparisons (GraphPad Prism, San Diego, CA, USA). Memory phenotypes were compared using a permutation test (10,000 rounds) in SPICE version 6.0 (http://exon.niaid.nih.gov/spice/).

## Results

### IN Vaccination With MCMV-M2 Elicits More Lung-Resident M2-Specific CD8^+^ T Cells Than IP Vaccination

**Figure 1:**
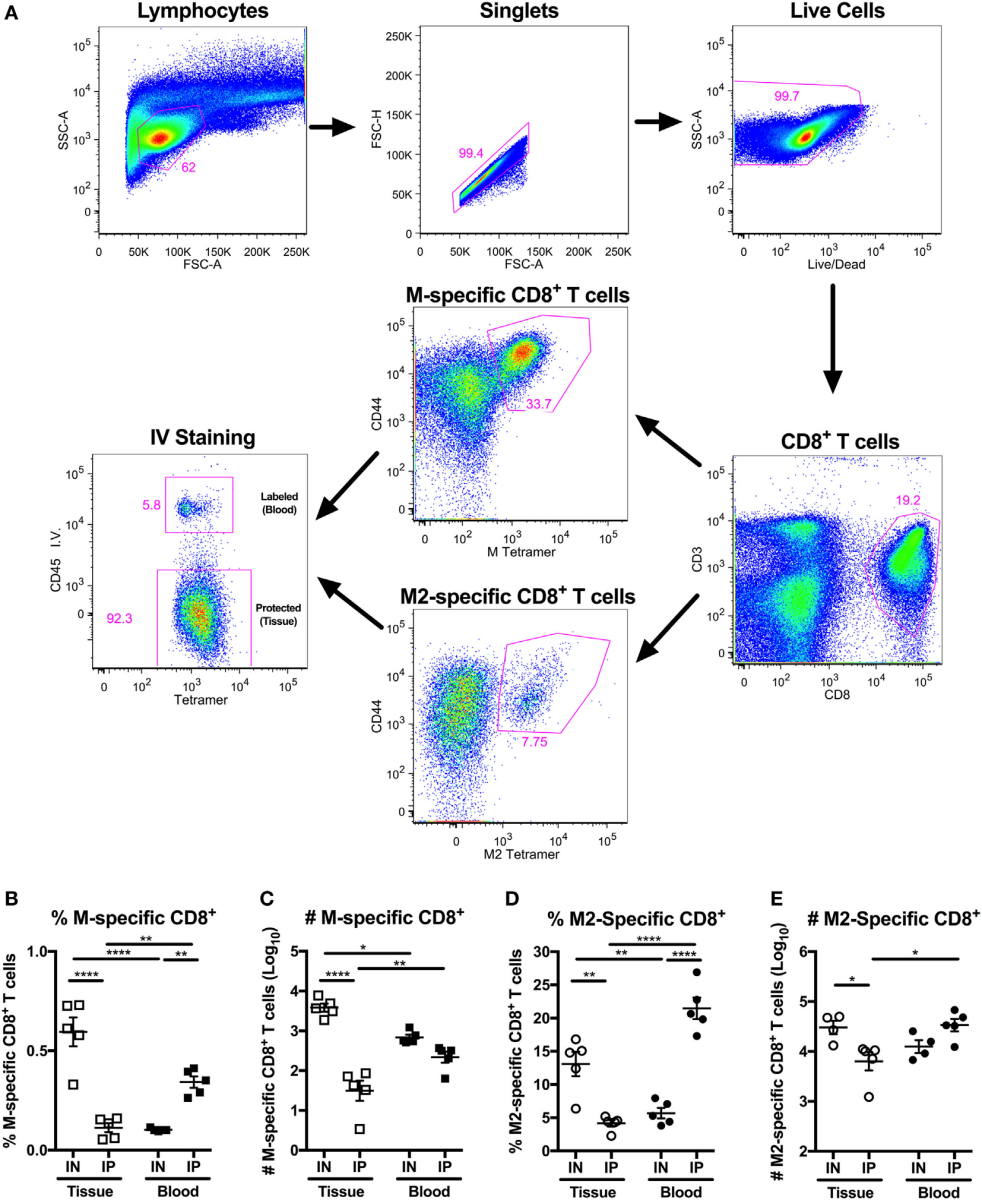
Intranasal (IN) vaccination with murine cytomegalovirus (MCMV)-M2 elicits more lung-resident M2-specific CD8^+^ T cells than intraperitoneal (IP) vaccination. **(A–E)** Mice were vaccinated with MCMV-M or MCMV-M2 *via* the IN or IP route. Intravascular staining was used in conjunction with D^b^/M_187–195_ and K^d^/M2_82–90_ tetramers to quantify epitope-specific CD8^+^ T cells in the lung tissue and blood after 1 week. **(A)** Gating strategy used to identify M-specific and M2-specific CD8^+^ T cells in the tissue and blood of the lungs. **(B)** Frequency and **(C)** number of M-specific CD8^+^ T cells in the tissue and blood of lungs 1 week after MCMV-M vaccination. **(D)** Frequency and **(E)** number of M2-specific CD8^+^ T cells in the tissue and blood of the lungs 1 week after MCMV-M2 vaccination. Bars indicate mean ± SEM (*n* = 5 mice/group). ****P < 0.0001, **P < 0.01, *P < 0.05 by two-way ANOVA. Data are shown from one experiment and representative of two independent experiments.

We and others have demonstrated that IN vaccination is necessary to elicit T_RM_ cells in the lungs (19, 23, 60). In particular, our earlier work showed that IN vaccination with MCMV-M elicited more M-specific CD8^+^ T cells in the lung parenchyma than IP vaccination with MCMV-M (Figure 1A) (60). To extend this finding, we vaccinated mice with MCMV-M2 *via* the IN or IP route and used intravascular staining in conjunction with K^d^/M2_82–90_ tetramers to analyze M2-specific CD8^+^ T cell responses in the blood and the lung parenchyma after 1 week. The intravascular staining protocol was validated in the context of IN vaccination to ensure that direct infection of the lungs did not lead to increased permeability due to inflammation (Figure S1 in Supplementary Material). Akin to the differences observed after vaccination with MCMV-M (Figures 1B,C), we found that IN vaccination with MCMV-M2 induced significantly more lung-resident M2-specific CD8^+^ T cells than IP vaccination with MCMV-M2, both in terms of frequency (*P* < 0.01; Figure 1D) and number (*P* < 0.05; Figure 1E). By contrast, IP vaccination with MCMV-M2 elicited higher frequencies of M2-specific CD8^+^ T cells in the blood (*P* < 0.0001, Figure 1D) and in total (*P* < 0.05), but similar numbers of M2-specific CD8^+^ T cells in the blood and in total. We therefore focused on IN vaccination in our efforts to induce and maintain lung-resident CD8^+^ T cells.

### The M-Specific CD8^+^ T Cell Population Inflates, Whereas the M2-Specific CD8^+^ T Cell Population Contracts, After Vaccination With MCMV

**Figure 2:**
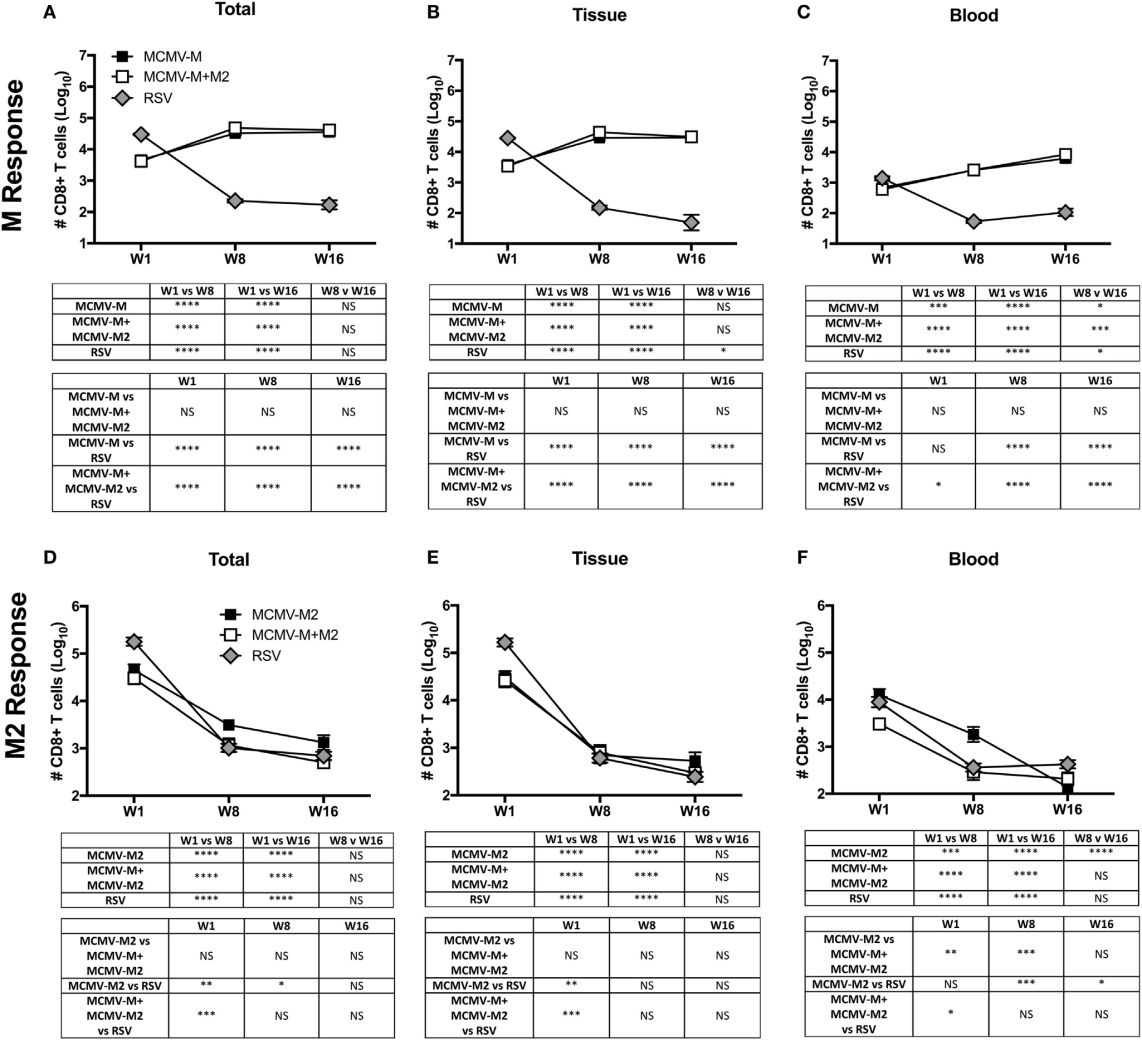
The M-specific CD8^+^ T cell population inflates, whereas the M2-specific CD8^+^ T cell population contracts, after vaccination with murine cytomegalovirus (MCMV). **(A–F)** Mice were infected with respiratory syncytial virus (RSV) or vaccinated with MCMV-M or MCMV-M2 alone or a combination of MCMV-M and MCMV-M2 *via* the intranasal route. Intravascular staining was used in conjunction with D^b^/M_187–195_ and K^d^/M2_82–90_ tetramers to quantify M-specific **(A–C)** and M2-specific **(D–F)** CD8^+^ T cells in the lung tissue and blood at weeks 1 (W1), 8 (W8), and 16 (W16). Total **(A,D)** denotes all tetramer^+^ CD8^+^ T cells regardless of location. Bars indicate mean ± SEM (*n* = 5 mice/group). ****P < 0.0001, ***P < 0.001, **P < 0.01, *P < 0.05 by two-way ANOVA. Data are shown from one experiment and representative of two independent experiments.

Next, we used a similar approach to evaluate CD8^+^ T cell responses at weeks 1, 8, and 16 after vaccination with MCMV-M or MCMV-M2 alone or a combination of MCMV-M and MCMV-M2. Intravascular staining was used as above in conjunction with D^b^/M_187–195_ and K^d^/M2_82–90_ tetramers to quantify epitope-specific CD8^+^ T cells in the blood and lung parenchyma. MCMV-M administered either alone or together with MCMV-M2 generated an M-specific CD8^+^ T cell population that inflated between weeks 1 and 8 (*P* < 0.0001) and remained stable through week 16 (Figure 2A). This trend was observed in the lung tissue and blood (*P* < 0.0001; Figures 2B,C). By contrast, M2-specific CD8^+^ T cells in the lung tissue and blood contracted over time (*P* < 0.0001; Figures 2D–F), irrespective of coadministration with MCMV-M. After RSV infection, which generates only conventional memory responses as a consequence of self-limited antigen production, the M-specific and M2-specific CD8^+^ T cell populations both contracted dramatically between weeks 1 and 8 in the lung tissue and blood (*P* < 0.001; Figures 2A–F). Similar epitope-specific patterns were observed when assessing T cell frequency in the lung and spleen (Figure S2 in Supplementary Material). In addition, the MCMV-encoded M38-specific CD8^+^ T cell response was largely equivalent among experimental groups, suggesting that the observed loss of M2-specific CD8^+^ T cells over time was not attributable to clearance of MCMV-M2. Thus, the M-specific CD8^+^ T cell population is inflationary, whereas the M2-specific CD8^+^ T cell population is not inflationary, after vaccination with MCMV.

One week after vaccination, coadministration of MCMV-M and MCMV-M2 elicited an epitope-specific hierarchy equivalent to that observed after RSV infection, with a dominant CD8^+^ T cell response to K^d^/M2_82–90_ and a subdominant CD8^+^ T cell response to D^b^/M_187–195_ (Figures 2A,D). At weeks 8 and 16, this hierarchy was inverted as a consequence of M-specific CD8^+^ T cell inflation and M2-specific CD8^+^ T cell contraction (Figures 2A,D). Coadministration of MCMV-M and MCMV-M2 did not alter the number or frequency of M-specific CD8^+^ T cells in the blood or the tissue at any time point relative to vaccination with MCMV-M alone (Figures 2B,C; Figure S2B,C in Supplementary Material). By contrast, coadministration of MCMV-M and MCMV-M2 dampened the frequency, but not the overall magnitude, of the M2-specific CD8^+^ T cell response at week 1 (*P* < 0.01), but not at weeks 8 and 16 (Figure 2D; Figure S2D in Supplementary Material). This effect was anatomically discrepant. Specifically, coadministration of MCMV-M and MCMV-M2 did not significantly reduce the number or frequency of M2-specific CD8^+^ T cells in the lung tissue (Figure 2E; Figure S2E in Supplementary Material), but did significantly reduce the number and frequency of M2-specific CD8^+^ T cells in the blood at weeks 1 and 8 relative to vaccination with MCMV-M2 alone (*P* < 0.01; Figure 2F; Figure S2F in Supplementary Material). No significant differences in the frequency of M2-specific CD8^+^ T cells were observed after contraction of the response at week 16 (Figures 2D–F). The reduction of M2-specific CD8^+^ T cells at the acute time point after coadministration of MCMV-M and MCMV-M2 was not unexpected, because competition between the M-specific and M2-specific CD8^+^ T cell populations has been demonstrated after RSV infection of CB6F1 mice (57).

### The M2 Epitope Is Preferentially Generated by the Immunoproteasome

**Figure 3:**
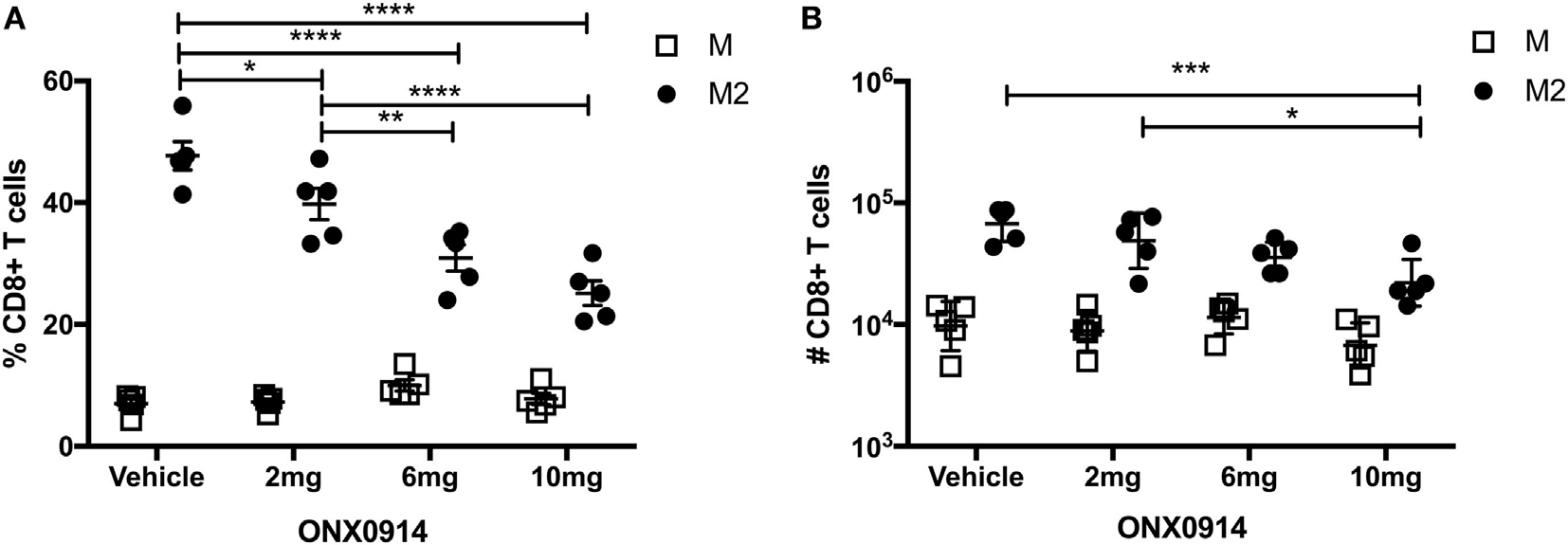
The M2 epitope is preferentially generated by the immunoproteasome. **(A,B)** Mice were infected with respiratory syncytial virus (RSV) and treated with the immunoproteasome inhibitor ONX-0914 or vehicle control on days 0, 2, 4, and 6 at doses of 2, 6, or 10 mg/kg. D^b^/M_187–195_ and K^d^/M2_82–90_ tetramers were used to quantify the frequency **(A)** and number **(B)** of M-specific and M2-specific CD8^+^ T cells in the lungs on day 7. ****P < 0.0001, ***P < 0.001, **P < 0.01, *P < 0.05 by two-way ANOVA. Bars indicate mean ± SEM (*n* = 5 mice/group). Data are shown from one experiment and representative of two independent experiments.

Memory inflation likely requires epitope generation *via* the constitutive proteasome, because antigen processing and presentation are thought to occur predominantly by non-hematopoietic cells, which lack immunoproteasomes (43, 44). To determine if proteasomal processing impacted the M-specific or M2-specific CD8^+^ T cell responses, we infected mice with RSV and treated them with the immunoproteasome inhibitor ONX-0914 on days 0, 2, 4, and 6 at doses of 2, 6, or 10 mg/kg. On day 7, we evaluated M-specific and M2-specific CD8^+^ T cells in the lungs. Treatment with ONX-0914 significantly reduced the frequency and number of M2-specific CD8^+^ T cells, but not M-specific CD8^+^ T cells, in a dose-dependent manner (Figures 3A,B). These data suggest that the M2 peptide is preferentially generated by the immunoproteasome, whereas the M peptide is preferentially generated by the constitutive proteasome, which is unaffected by ONX-0914.

### IN Vaccination With MCMV Elicits CD8^+^ T_RM_ Cells

**Figure 4:**
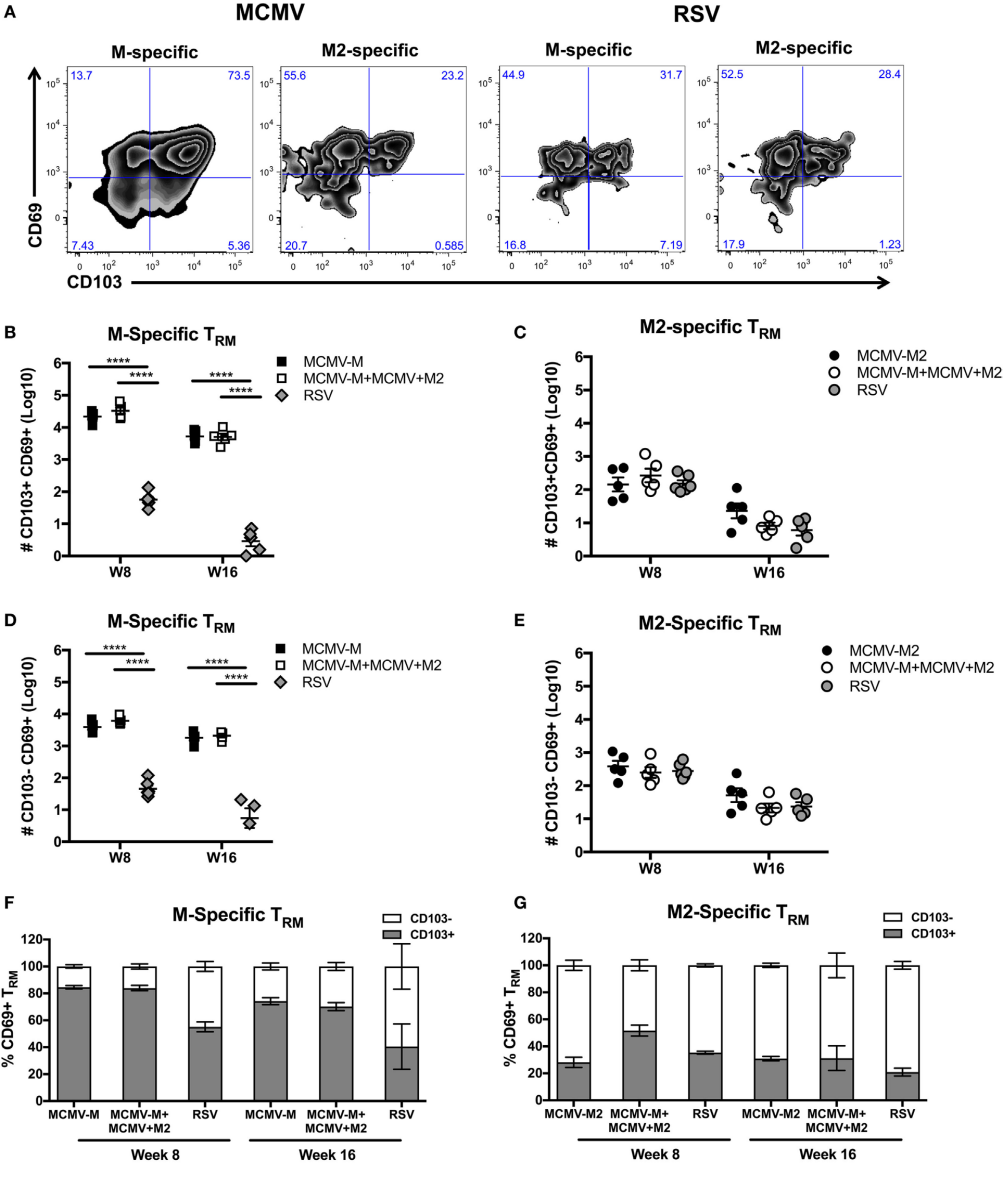
Intranasal (IN) vaccination with murine cytomegalovirus (MCMV) elicits CD8^+^ tissue-resident memory T (T_RM_) cells. **(A–E)** Mice were infected with respiratory syncytial virus (RSV) or vaccinated with MCMV-M or MCMV-M2 alone or a combination of MCMV-M and MCMV-M2 *via* the IN route. Intravascular staining was used in conjunction with D^b^/M_187–195_ and K^d^/M2_82–90_ tetramers to quantify M-specific **(A,B,D,F)** and M2-specific **(A,C,E,G)** CD8^+^ T cells in the lung tissue at weeks 8 (W8) and 16 (W16). **(A)** Representative flow cytometry plots showing expression of CD69 and CD103 on epitope-specific CD8^+^ T cells in the lung parenchyma at week 8. **(B,D)** The number of M-specific CD103^+^CD69^+^ T_RM_ cells **(B)** and CD103^−^CD69^+^ T_RM_ cells **(D)** elicited by infection with RSV or vaccination with MCMV-M alone or together with MCMV-M2. **(C,E)** The number of M2-specific CD103^+^CD69^+^ T_RM_ cells **(C)** and CD103^−^CD69^+^ T_RM_ cells **(E)** elicited by infection with RSV or vaccination with MCMV-M2 alone or together with MCMV-M. **(F,G)** Percentage of CD103^+^ and CD103^−^ M-specific CD69^+^ T_RM_ cells **(F)** and M2-specific CD69^+^ T_RM_ cells **(G)**. ****P < 0.0001 by two-way ANOVA. Bars indicate mean ± SEM (*n* = 5 mice/group). Data are shown from one experiment and representative of two independent experiments.

A previous study demonstrated that IN vaccination with MCMV-M generated a robust population of T_RM_ cells, identified by expression of CD103 (60). However, it has also been shown that not all T_RM_ cells express CD103 (7). We therefore used intravascular staining to quantify M-specific and M2-specific T_RM_ cells in the lung parenchyma based on expression of CD69 and CD103 after infection with RSV or vaccination with MCMV-M or MCMV-M2 alone or a combination of MCMV-M and MCMV-M2. The administration of MCMV-M, either alone or together with MCMV-M2, generated a substantial population of CD69^+^ T_RM_ cells that was largely maintained between weeks 8 and 16, and significantly outnumbered the corresponding population of CD69^+^ T_RM_ cells induced by RSV infection at both time points (*P* < 0.0001; Figures 4A,B,D). By contrast, the M2-specific CD69^+^ T_RM_ population significantly decreased between weeks 8 and 16, irrespective of M2 protein expression *via* MCMV or RSV (*P* < 0.01; Figures 4A,C, E). There was no difference in the number of M2-specific T_RM_ cells elicited by vaccination with MCMV-M2 or infection with RSV. As the M-specific T_RM_ population induced by MCMV was maintained in the lungs and the M2-specific T_RM_ population induced by MCMV waned in the lungs, there were significantly more M-specific T_RM_ cells than M2-specific T_RM_ cells in the lung parenchyma at weeks 8 and 16 (*P* < 0.0001; Figures 4B–E). In the context of RSV infection, however, there were significantly more M2-specific T_RM_ cells than M-specific T_RM_ cells in the lung parenchyma at both time points (*P* < 0.05; Figures 4B–E). Coadministration of MCMV-M and MCMV-M2 did not affect the number of M-specific or M2-specific T_RM_ cells at either time point compared with the administration of MCMV-M or MCMV-M2 alone (Figures 4B–E).

Next, we assessed the expression of CD103 on CD69^+^ T_RM_ cells. After vaccination with single MCMV vectors, a higher proportion of M-specific CD8^+^ T cells coexpressed CD69 and CD103 compared with M2-specific cells at week 8 (84.6 vs. 28.1%; *P* < 0.0001) and week 16 (74.2 vs. 30.9%; *P* < 0.05) (Figures 4F,G). A similar trend was observed after coadministration of MCMV-M and MCMV-M2. At week 8, the vaccine-induced M-specific T_RM_ population also contained a significantly higher proportion of cells expressing CD103 than the M-specific T_RM_ population elicited by RSV infection (84.6% for MCMV-M and 83.9% for MCMV-M + MCMV-M2 vs. 55% for RSV; *P* < 0.0001).

These data show that inflation of the M-specific CD8^+^ T cell population elicited by vaccination with MCMV enhances the frequency and number of T_RM_ cells relative to acute infection with RSV. By contrast, M2-specific CD8^+^ T_RM_ cells were induced at similar levels irrespective of M2 protein expression *via* MCMV or RSV. It is also notable that a larger fraction of M-specific CD69^+^ T_RM_ cells elicited by vaccination with MCMV coexpressed CD103 compared with either M2-specific CD69^+^ T_RM_ cells elicited by vaccination with MCMV or T_RM_ cells of either specificity elicited by infection with RSV.

### M-Specific and M2-Specific CD8^+^ T Cells Are Phenotypically Distinct in the Lung Tissue and Blood After Vaccination With MCMV

**Figure 5:**
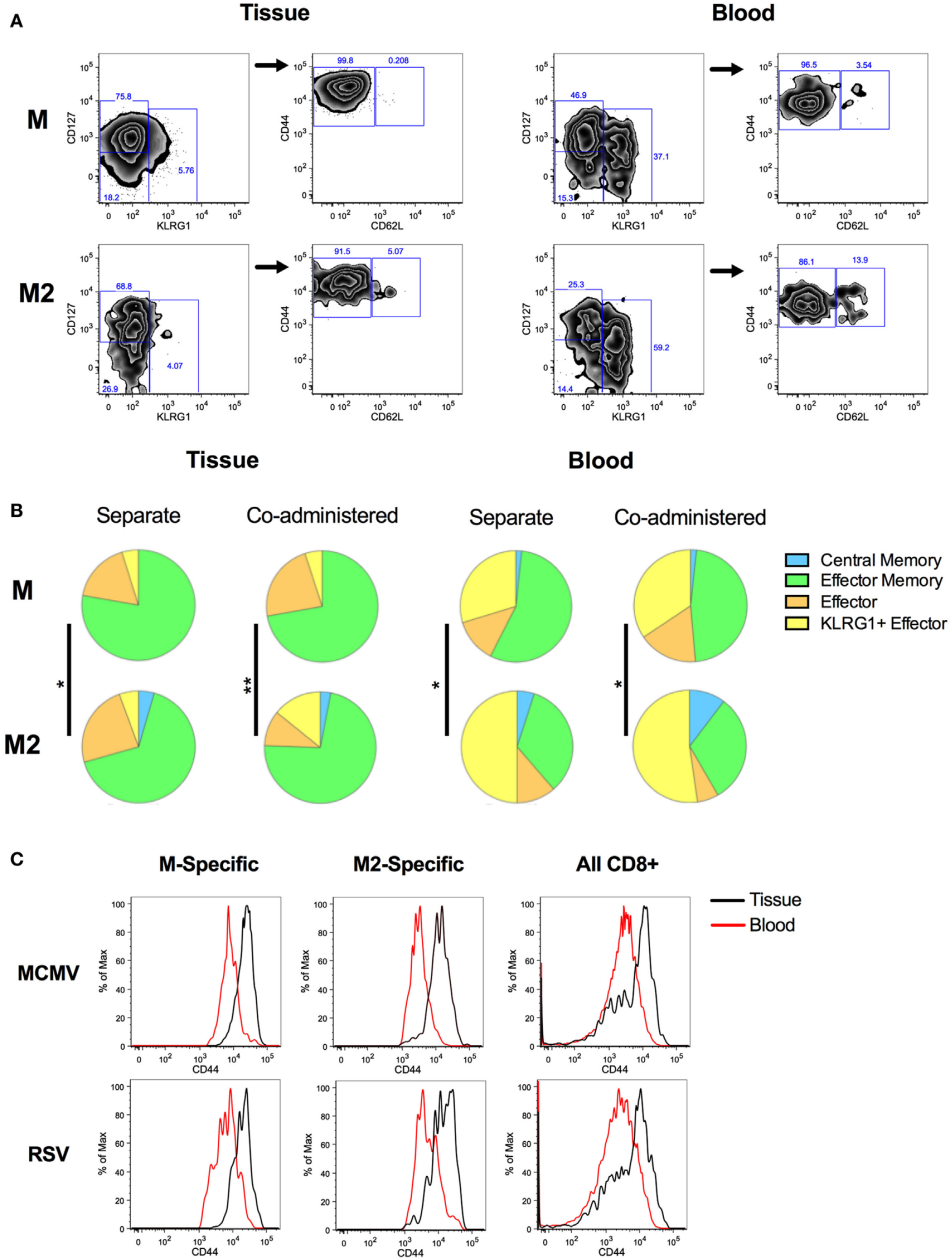
Phenotype of M-specific and M2-specific CD8^+^ T cells elicited by murine cytomegalovirus (MCMV) vaccination. Mice were vaccinated with MCMV-M or MCMV-M2 alone or a combination of MCMV-M and MCMV-M2 *via* the IN route. Intravascular staining was used in conjunction with D^b^/M_187–195_ and K^d^/M2_82–90_ tetramers to identify M-specific and M2-specific CD8^+^ T cells in the blood and tissue of the lungs at week 8. **(A)** Gating strategy for phenotypic analysis. Populations were defined as follows: CD127^+^KLRG1^−^CD62L^+^ [central memory (CM)]; CD127^+^KLRG1^−^CD62L^−^ [effector memory (EM)]; CD127^−^KLRG1^−^CD62L^−^ (effector); and KLRG1^+^CD62L^−^ (KLRG1^+^ effector). **(B)** The proportions of CM cells (blue), EM cells (green), effectors (orange), and KLRG1^+^ effectors (yellow) in the lungs are shown for each specificity. **(C)** CD44 expression on M-specific, M2-specific, and all CD8^+^ T cells in the tissue and blood of the lungs. *P ≤ 0.05, **P < 0.01 by permutation test (SPICE). Data are shown from one experiment (*n* = 5/group) and representative of two independent experiments.

Inflationary and conventional epitope-specific CD8^+^ T cell populations have previously been shown to differ phenotypically after IP infection with MCMV (63). In this context, inflationary memory cells are predominantly CD127^−^KLRG1^+^ effectors, while conventional memory cells display a more CD127^+^CD62L^+^ central memory (CM)-like phenotype. This pattern is recapitulated after IP vaccination with MCMV-M. However IN vaccination with MCMV-M induces a CD8^+^ T cell population with predominantly effector and EM phenotypes (60). We therefore analyzed the phenotype of antigen-specific CD8^+^ T cells elicited by MCMV-M and/or MCMV-M2 vaccination at 8 weeks post-vaccination. We categorized the RSV-specific CD8^+^ T cell populations as CM, EM, effector, or KLRG1^+^ effectors (KLRG1^+^) (Figure 5). Populations were defined as follows: all: tetramer^+^ CD44^+^; CM: CD127^+^KLRG1^−^CD62L^+^; EM: CD127^+^KLRG1^−^CD62L^−^; effector: CD127^−^KLRG1^−^CD62L^−^; KLRG1^+^ effector: CD62L^−^KLRG1^+^. Overall, there were no obvious phenotypic differences when the MCMV vectors were administered alone or in combination (Figure 5B). By contrast, distinct phenotypes were observed across anatomical compartments for both the M-specific and M2-specific CD8^+^ T cell populations, with higher frequencies of KLRG1^+^ effectors (yellow) and CM cells (blue) and lower frequencies of EM cells (green) in the blood compared with the tissue (*P* < 0.05). A comparison of M-specific and M2-specific CD8^+^ T cells in the blood and tissue also showed that these antigen-specific populations were comprised of different proportions of memory subsets (Figure 5, *P* < 0.05). In the blood, the M2-specific CD8^+^ T cell population incorporated larger fractions of CM (blue) and KLRG1^+^ effectors (yellow) and smaller fractions of effector (orange) and EM (green) cells than the M-specific CD8^+^ T cell population. Although statistically significant, the differences between the M-specific and M2-specific CD8^+^ T cell population were more subtle in the tissue. Interestingly, we observed higher levels of CD44 expression on CD8^+^ T cells in the lung tissue compared with CD8^+^ T cells in the blood, irrespective of antigen specificity and vaccination modality (Figure 5C). When parsed out by location, expression of CD44 by M-specific and M2-specific CD8^+^ T cells was relatively high compared with the corresponding bulk CD8^+^ T cell populations in the blood and tissue of the lungs (Figure S3 in Supplementary Material).

### MCMV-Elicited T_RM_ Cells Expedite Viral Clearance After Infection With RSV

**Figure 6:**
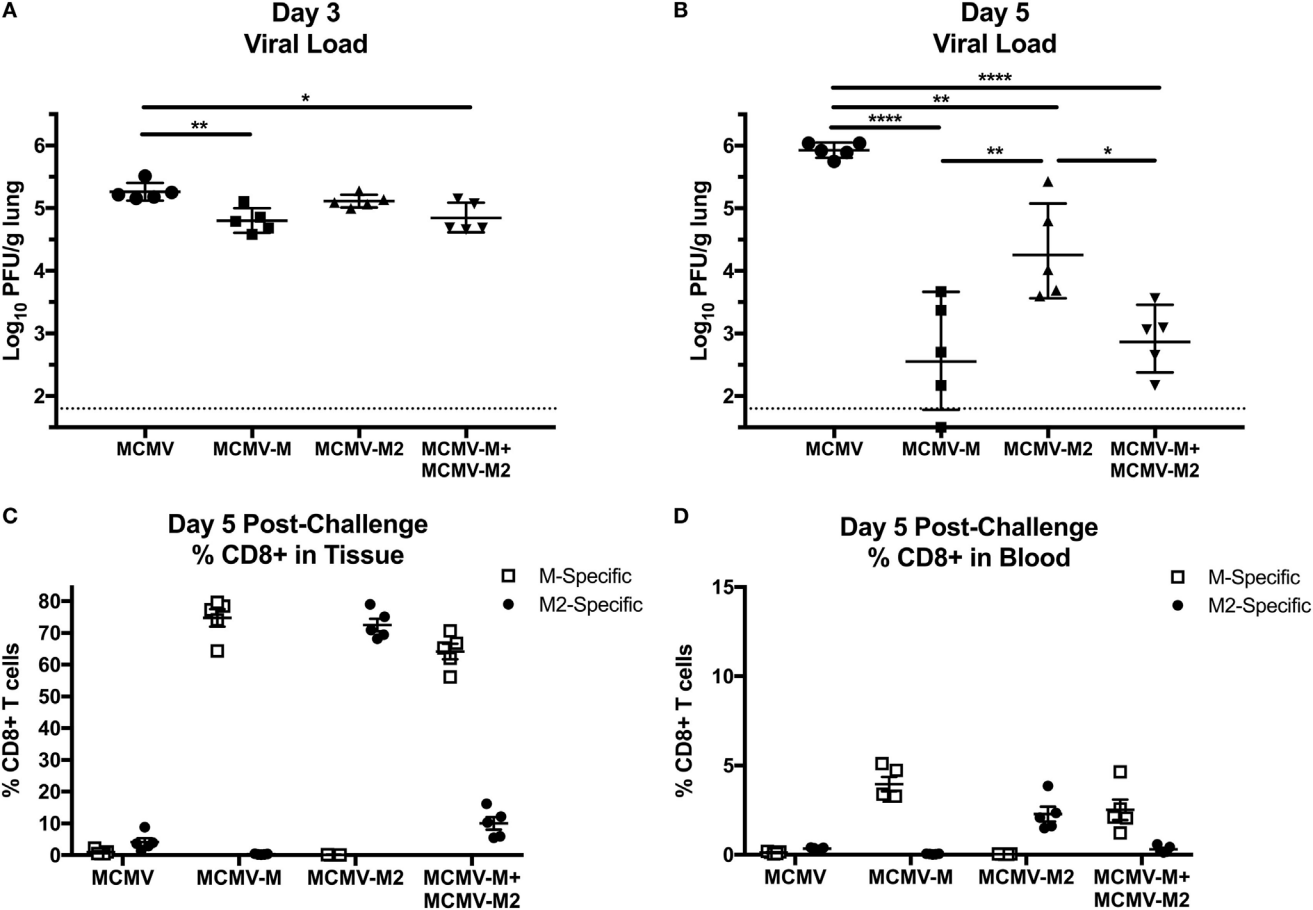
Murine cytomegalovirus (MCMV)-elicited tissue-resident memory T cells expedite viral clearance after infection with respiratory syncytial virus (RSV). **(A–D)** Mice were vaccinated with MCMV vector, MCMV-M or MCMV-M2 alone, or a combination of MCMV-M and MCMV-M2 *via* the intranasal route and challenged with RSV at week 16. Viral titers in the lungs were measured by plaque assay on days 3 **(A)** and 5 **(B)**. ****P < 0.0001, ***P < 0.001, **P < 0.01, *P < 0.05 by one-way ANOVA. **(C,D)** Intravascular staining was used in conjunction with D^b^/M_187–195_ and K^d^/M2_82–90_ tetramers to quantify M-specific and M2-specific CD8^+^ T cells in the lungs **(C)** and the blood **(D)**. ****P < 0.0001, **P < 0.01, *P < 0.05 by two-way ANOVA. Data shown from one experiment and representative of two independent experiments.

To evaluate the biological relevance of these observations, we challenged mice with 2 × 10^6^ PFU of RSV delivered *via* the IN route 16 weeks after vaccination with MCMV-M, MCMV-M2, or a combination of MCMV-M and MCMV-M2. Viral loads were measured on days 3 and 5 after infection with RSV. On day 3, mice vaccinated with MCMV-M or MCMV-M together with MCMV-M2 exhibited significantly lower viral loads in the lungs compared with mice vaccinated with the MCMV vector alone (*P* < 0.01 and *P* < 0.05, respectively; Figure 6A). By contrast, vaccination with MCMV-M2 did not lead to a significant reduction in viral load on day 3. All vaccination regimens significantly reduced viral loads on day 5 relative to the MCMV vector alone (*P* < 0.0001 for MCMV-M, *P* < 0.01 for MCMV-M2, *P* < 0.0001 for MCMV-M + MCMV-M2; Figure 6B). However, simultaneous vaccination with MCMV-M and MCMV-M2 did not enhance viral clearance relative to vaccination with MCMV-M alone (Figure 6B).

### Inflation of the M-Specific CD8^+^ T Cell Population Alters Immunodominance After Challenge With RSV

In further experiments, we assessed the frequency of antigen-specific CD8^+^ T cells in the lung parenchyma on day 5 after challenge with RSV (Figures 6C,D). Mice vaccinated with the MCMV vector alone harbored relatively few M-specific or M2-specific CD8^+^ T cells in the lungs, but the M2-specific population was immunodominant, as typically observed in unvaccinated mice after infection with RSV. As expected, mice vaccinated with MCMV-M or MCMV-M2 alone mounted immunodominant CD8^+^ T cell responses to the corresponding vaccine antigens, whereas mice vaccinated with both MCMV-M and MCMV-M2 displayed very high frequencies of M-specific CD8^+^ T cells relative to M2-specific CD8^+^ T cells, inverting the natural immunodominance hierarchy observed after infection with RSV. This finding may explain why the addition of MCMV-M2 did not enhance the protective effects of vaccination with MCMV-M alone in response to challenge with RSV.

## Discussion

Vaccination with MCMV-M *via* the IN route has been shown to generate a robust population of M-specific CD8^+^ T_RM_ cells in the lungs that subsequently inflates over time (60). To extend this finding, we evaluated MCMV vaccine-induced CD8^+^ T cell responses to the immunodominant M2 epitope. We found that IN vaccination with MCMV-M2 induced a conventional memory response, but failed to establish a stable population of lung-resident M2-specific CD8^+^ T_RM_ cells. Moreover, coadministration of MCMV-M and MCMV-M2 inverted the natural immunodominance hierarchy, but did not significantly impact the generation of M-specific or M2-specific CD8^+^ T_RM_ cells. As a consequence, the protective effects of vaccination with MCMV-M were neither impeded nor enhanced by the addition of MCMV-M2.

Memory inflation is essential for the maintenance of lung-resident CD8^+^ T_RM_ cell populations. In the setting of self-limiting viral infections of the respiratory tract, conventional epitopes induce populations of CD8^+^ T_RM_ cells in the lung parenchyma that wane over time (14). Our data further show that persistent antigen expression is insufficient to overcome this decline, consistent with the findings of Smith et al., who demonstrated that T_RM_ cells are maintained in the salivary glands *via* continuous production rather than *via* long-term survival after infection with MCMV (26). In our previous work, we demonstrated that the robust population of M-specific CD8^+^ T_RM_ cells induced by IN vaccination with MCMV-M contributed to early clearance of RSV (60). This effect was maintained after treatment with a sphingosine 1-phosphate receptor modulator, suggesting that protection was independent of recirculation *via* the lymph nodes. These data concur with the observation herein that IN vaccination with MCMV-M2 failed to mediate early immune control of RSV. Together, these studies highlight the importance of lung-tropic T_RM_ cells in protection against respiratory infection. Accordingly, immunization with a persistent vector offers no immediate advantages over traditional vaccine platforms for conventional epitopes like M2. By contrast, the induction and maintenance of inflationary epitope-specific CD8^+^ T_RM_ cells in the lungs after vaccination with MCMV may enhance immune protection against respiratory pathogens, which typically induce only transient memory responses at the site of infection (14–16, 24).

Several factors determine the immunogenicity and memory characteristics of any given epitope. In this study, the M and M2 sequences were inserted into the IE2 gene, which naturally encodes inflationary epitopes, and the proteins were under the control of the constitutive promoter IE1 (30, 31, 45). Despite identical genomic locations, the M2 epitope failed to elicit inflationary CD8^+^ T cell responses. This lack of inflation may reflect greater dependence on the immunoproteasome compared with the M-specific CD8^+^ T cell response, consistent with previous studies that postulated a key role for antigen processing as a determinant of immunodominance patterns in the context of infection with MCMV (43, 44). In addition, M-specific CD8^+^ T cells operate with higher composite avidities than M2-specific CD8^+^ T cells after infection with RSV (57). However, this factor alone may not preclude M2-driven memory inflation, because recent work has demonstrated the existence of low-avidity inflationary CD8^+^ T cell populations (41). It is also difficult to exclude other possible influences, such as competition between CD8^+^ T cells with different antigen specificities and variable requirements for co-stimulation and CD4^+^ T cell help, which are more difficult to assess directly. Any or all of these factors may contribute to the lack of inflation among M2-specific CD8^+^ T cells. *In vivo* testing is therefore required to assess the true inflationary potential of any given epitope, a process that will become more difficult as vaccines advance from inbred animal models to human populations with diverse genetic backgrounds. A better understanding of the factors that govern memory inflation and how they can be manipulated will be important for the development of CMV vaccines.

As memory inflation is difficult to predict, it is important to study the effect of both inflationary and conventional epitopes in vaccine settings. Coadministration of MCMV-M and MCMV-M2 reduced the overall magnitude of the conventional M2-specific CD8^+^ T cell response acutely after vaccination but did not impact the inflationary M-specific CD8^+^ T cell response at any stage after vaccination. Moreover, dual immunization was equivalent to vaccination with MCMV-M alone in terms of protective efficacy after challenge with RSV. These data suggest that both conventional and inflationary epitopes can be included in a persistent vaccine without detrimental effects. However, it should be noted that competition for antigen can occur if inflationary epitopes are delivered by the same vector (45). Individual epitopes are therefore probably best expressed separately if polyvalency is required to prevent immune escape.

In summary, we have shown that memory inflation is required for the maintenance of CD8^+^ T_RM_ cells in the lungs after IN vaccination with MCMV. These findings highlight an important consideration in the development of persistent vectors and suggest that epitope selection will be a central determinant of efficacy in the setting of vaccines that deliver antigens on a continuous basis.

## Ethics Statement

This study was carried out in accordance with the recommendations and guidelines of the NIH Guide to the Care and Use of Laboratory Animals. The protocol was approved by the Animal Care and Use Committee of the Vaccine Research Center, National Institute of Allergy and Infectious Diseases, National Institutes of Health. Mice were housed in a facility fully accredited by the Association for Assessment and Accreditation of Laboratory Animal Care International (AAALAC). Animal procedures were conducted in strict accordance with all relevant federal and National Institutes of Health guidelines and regulations.

## Author Contributions

KM, TR, DP, and BG conceived and designed studies; KM, TR, EB-H, and DN performed animal studies and analyzed data; SM and AR designed and generated recombinant BACs; KM, TR, DP, and BG wrote the manuscript. All authors provided critical feedback and approved the manuscript.

## Conflict of Interest Statement

The authors declare that the research was conducted in the absence of any commercial or financial relationships that could be construed as a potential conflict of interest.
