# Supplementary material for: Memory Inflation Drives Tissue-Resident Memory CD8+ T Cell Maintenance in the Lung After Intranasal Vaccination With Murine Cytomegalovirus
Source: Front Immunol. 2018 Aug 14;9:1861. doi: 10.3389/fimmu.2018.01861 (PMC6102355; doi:10.3389/fimmu.2018.01861)
Supplement: Supplementary file 1 [file Data_Sheet_1.pdf]

Supplementary Data

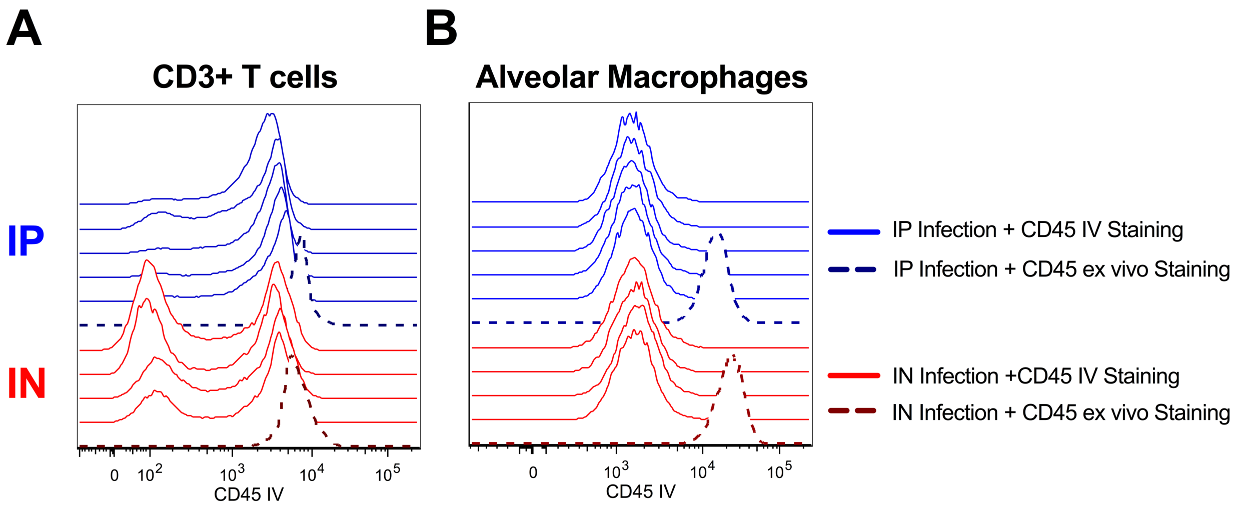

Supplementary Figure 1. Intravascular staining is not affected by Intranasal vaccination with MCMV. Mice were vaccinated with MCMV-M via the IN (red) or IP (blue) route. On day 7 post-vaccination, mice were injected IV with 3µg of anti-CD45-PerCP-Cy5.5 and euthanized 5 minutes later. Lungs were harvested and stained ex vivo for (**A**) T cells (CD3+) and (**B**) alveolar macrophages (CD3-CD11c^{+}CD64^{+}SiglecF^{+}). Lines represent individual mice with 4-5 mice/group. Lungs from one mouse/group were stained ex vivo for CD45 (dashed lines).

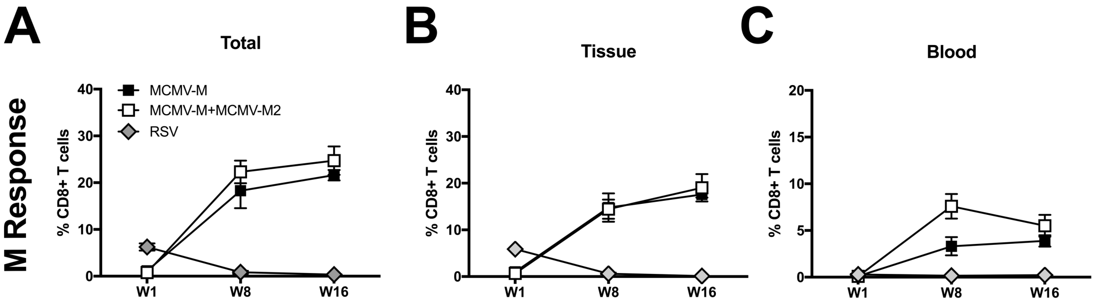

	W1 vs W8	W1 vs W16	W8 v W16
MCMV-M	****	****	NS
MCMV-M+ MCMV-M2	****	****	NS
RSV	NS	NS	NS

	W1	W8	W16
MCMV-M vs MCMV-M+ MCMV-M2	NS	NS	NS
MCMV-M vs RSV	NS	****	****
MCMV-M+ MCMV-M2 vs RSV	NS	****	****

	W1 vs W8	W1 vs W16	W8 v W16
MCMV-M	****	****	NS
MCMV-M+ MCMV-M2	****	****	NS
RSV	NS	*	NS

	W1	W8	W16
MCMV-M vs MCMV-M+ MCMV-M2	NS	NS	NS
MCMV-M vs RSV	NS	****	****
MCMV-M+ MCMV-M2 vs RSV	NS	****	****

	W1 vs W8	W1 vs W16	W8 v W16
MCMV-M	**	**	NS
MCMV-M+ MCMV-M2	****	****	NS
RSV	NS	NS	NS

	W1	W8	W16
MCMV-M vs MCMV-M+ MCMV-M2	NS	***	NS
MCMV-M vs RSV	NS	**	**
MCMV-M+ MCMV-M2 vs RSV	NS	****	****

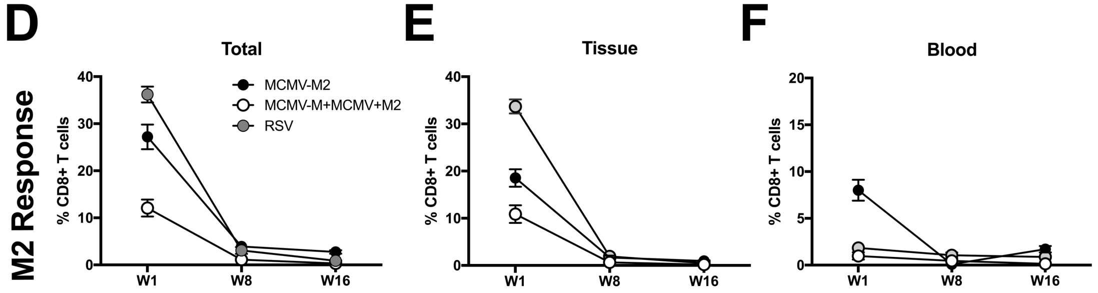

	W1 vs W8	W1 vs W16	W8 v W16
MCMV-M2	****	****	NS
MCMV-M+ MCMV-M2	****	****	NS
RSV	****	****	NS

	W1	W8	W16
MCMV-M2 vs MCMV-M+ MCMV-M2	****	NS	NS
MCMV-M2 vs RSV	****	NS	NS
MCMV-M+ MCMV-M2 vs RSV	****	NS	NS

	W1 vs W8	W1 vs W16	W8 v W16
MCMV-M2	****	****	NS
MCMV-M+ MCMV-M2	****	****	NS
RSV	****	****	NS

	W1	W8	W16
MCMV-M2 vs MCMV-M+ MCMV-M2	****	NS	NS
MCMV-M2 vs RSV	****	NS	NS
MCMV-M+ MCMV-M2 vs RSV	****	NS	NS

	W1 vs W8	W1 vs W16	W8 v W16
MCMV-M2	****	****	*
MCMV-M+ MCMV-M2	NS	NS	NS
RSV	NS	NS	NS

	W1	W8	W16
MCMV-M2 vs MCMV-M+ MCMV-M2	****	NS	NS
MCMV-M2 vs RSV	****	NS	NS
MCMV-M+ MCMV-M2 vs RSV	NS	NS	NS

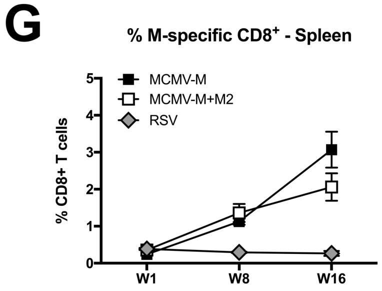

	W1 vs W8	W1 vs W16	W8 v W16
MCMV-M	*	****	****
MCMV-M+ MCMV-M2	**	****	NS
RSV	NS	NS	NS

	W1	W8	W16
MCMV-M vs MCMV-M+ MCMV-M2	NS	NS	NS
MCMV-M vs RSV	NS	*	**
MCMV-M+ MCMV-M2 vs RSV	**	****	****

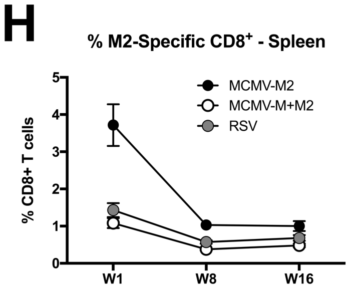

	W1 vs W8	W1 vs W16	W8 v W16
MCMV-M2	****	****	NS
MCMV-M+ MCMV-M2	NS	NS	NS
RSV	*	NS	NS

	W1	W8	W16
MCMV-M2 vs MCMV-M+ MCMV-M2	****	NS	NS
MCMV-M2 vs RSV	****	NS	NS
MCMV-M+ MCMV-M2 vs RSV	NS	NS	NS

Supplementary Figure 2. The M-specific $CD8^+$ T cell population inflates, whereas the M2-specific $CD8^+$ T cell population contracts, after vaccination with MCMV. (**A**–**F**) Mice were infected with RSV or vaccinated with MCMV-M or MCMV-M2 alone or a combination of MCMV-M and MCMV-M2 via the intranasal route. Intravascular staining was used in conjunction with $D^b/M_{187\text{-}195}$ and $K^d/M2_{82\text{-}90}$ tetramers to determine the frequency of M-specific (**A**–**C**) and M2-specific (**D**–**F**) $CD8^+$ T cells in the lung tissue and blood at weeks 1 (W1), 8 (W8), and 16 (W16). Total (**A, D**) denots all $tetramer^+$ $CD8^+$ T cells regardless of location. **(G,H)** Frequency of total M-specific (**G**) and M2-specific (H) $CD8^+$ T cells in the spleen. Bars indicate mean ± SEM (n = 5 mice/group). **** $P < 0.0001$, *** $P < 0.001$, ** $P < 0.01$, * $P < 0.05$ by two-way ANOVA. Data shown from one experiment and representative of two independent experiments.

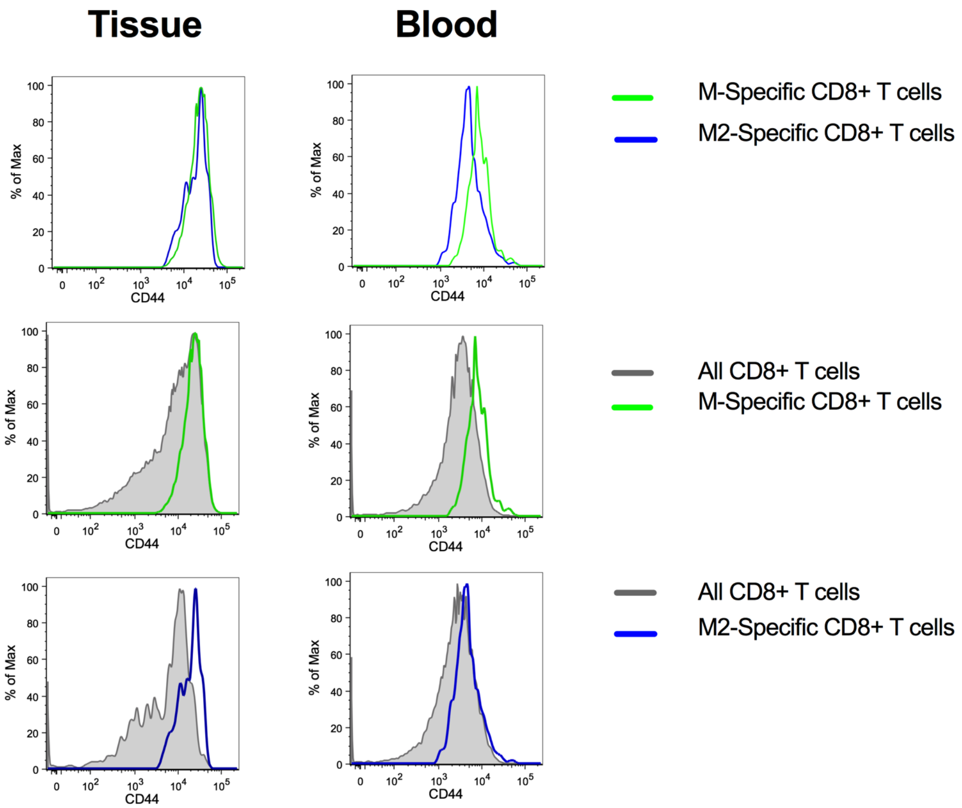

Supplementary Figure 3. Comparison of CD44 expression on M-specific and M2-specific $CD8^+$ T cells. CD44 expression was determined on M-specific (green), M2-specific (blue), and all (grey shaded) $CD8^+$ T cells at week 8 after vaccination with MCMV-M or MCMV-M2. Intravascular staining was used in conjunction with D^b/M$_{187-195}$ and K^d/M2$_{82-90}$ tetramers to identify M-specific and M2-specific $CD8^+$ T cells in the blood and tissue of the lungs. Data are representative two independent experiments (n = 5 mice/group).
